# Urotensin-related gene transcripts mark developmental emergence of the male forebrain vocal control system in songbirds

**DOI:** 10.1038/s41598-018-37057-w

**Published:** 2019-01-28

**Authors:** Zachary W. Bell, Peter Lovell, Claudio V. Mello, Ping K. Yip, Julia M. George, David F. Clayton

**Affiliations:** 1 Department of Biological and Experimental Psychology, School of Biological and Chemical Sciences, Queen Mary University of London, Mile End Road, London, E1 4NS UK; 2 Department of Behavioral Neuroscience, Oregon Health and Science University, 3181 Sam Jackson Park Road, L470, Portland, Oregon 97239-3098 USA; 3 Centre for Neuroscience & Trauma, Blizard Institute, Queen Mary University of London, Barts and the London School of Medicine and Dentistry, 4 Newark St, London, E1 2AT UK; 4 Present Address: Shoreditch-son Co., Ltd., 1919-1 Tancha, Onna, Kunigami District, Okinawa Prefecture 904-0412 Japan

## Abstract

Songbirds communicate through learned vocalizations, using a forebrain circuit with convergent similarity to vocal-control circuitry in humans. This circuit is incomplete in female zebra finches, hence only males sing. We show that the *UTS2B* gene, encoding Urotensin-Related Peptide (URP), is uniquely expressed in a key pre-motor vocal nucleus (HVC), and specifically marks the neurons that form a male-specific projection that encodes timing features of learned song. *UTS2B*-expressing cells appear early in males, prior to projection formation, but are not observed in the female nucleus. We find no expression evidence for canonical receptors within the vocal circuit, suggesting either signalling to other brain regions via diffusion or transduction through other receptor systems. Urotensins have not previously been implicated in vocal control, but we find an annotation in Allen Human Brain Atlas of increased *UTS2B* expression within portions of human inferior frontal cortex implicated in human speech and singing. Thus *UTS2B* (URP) is a novel neural marker that may have conserved functions for vocal communication.

## Introduction

Vocal learning ability is a distinctive trait shared by humans and songbirds and only a few other animal groups^1^. Songbirds thus provide an important animal model for defining common molecular and physiological features of brain systems needed for vocal learning. In both humans and songbirds, vocal learning is controlled by specialized circuits that share similar connections and molecular properties^2,3^. In songbirds, nucleus HVC (used as a proper name^4^) represents a central control point for learned vocalizations and can be considered an avian analogue of vocal premotor cortex, possibly analogous either to Broca’s area for speech control or to supragranular layers of the laryngeal motor cortex^3,5–8^. Neurophysiological activity in HVC provides timing cues considered important for driving the production of highly stereotyped learned vocal sequences^9–12^. Nucleus HVC and connected forebrain vocal learning nuclei are absent in bird species that lack vocal learning ability, and HVC shows marked sex differences in size and anatomy that parallel sex differences in vocal behaviour^13–17^. In the male zebra finch, HVC begins to emerge as an anatomically distinct nucleus about two weeks after hatching^18–21^, and ultimately forms two major projections: one to striatal area X, necessary for learning of vocalizations, and a second to vocal motor robust nucleus of the arcopallium (RA), necessary for production of learned vocalizations^22^. The projection from HVC to RA does not develop in female zebra finches^17^ unless they have been treated with gonadal steroids in the first few weeks after hatching^23–25^. The physiological, anatomical, developmental and molecular properties of HVC have been subjects of continuing interest, and several hundred genes have now been identified as local markers of the nucleus based on high-throughput microarray hybridization data^26^.

Urotensin II (*UTS2* gene) and its paralog URP (Urotensin related protein, *UTS2B* gene also known as *UTS2D*; see Table 1) are potent vasoactive peptides which exert biological effects through a canonical family of G protein-coupled receptors (urotensin II receptors, formerly GPR14 orphan receptors^27^). Functionally, the urotensinergic system in other species has been implicated in cardiovascular, renal and immune regulation (reviewed^28^). Intriguingly, the *UTS2B* RNA is enriched in songbird HVC^26,29^, and also reported to be up-regulated there after androgen treatments that promote singing and growth of HVC^29,30^. The potential involvement of urotensin peptides in vocal control, or indeed in higher telencephalic functions in general, is without precedent in studies of other vertebrates: in both rodents and fish, the orthologue is expressed mainly in brainstem nuclei and spinal cord^28^, and little or no expression has been described in the telencephalon^31^.

**Table 1:** Nomenclature of Urotensinergic System Genes and Peptides in the Zebra Finch, based on Entrez Gene IDs and the receptor nomenclature of Tostivint^35^.

Vertebrate Nomenclature	Peptide	Synonyms	Gene ID (chicken)	Gene Symbol (chicken)	Gene ID (zebra finch)	Assigned Gene Symbol (zebra finch)
*UTS2*	UII	Urotensin-II	404535	*UTS2*	100222633	*UTS2*
*UTS2B*	URP	*UTS2*D; Urotensin-related peptide; Urotensin-IIB peptide; Urotensin-II related peptide.	404534	*UTS2B*	100218300	*UTS2B*
*UTS2R1*		Urotensin-2 Receptor	427800	*UTS2R*	100231339	*UTS2R1*
*UTS2R2*		Urotensin-2 Receptor-like	101747873	*LOC101747873*	NA	*UTS2R2*P
*UTS2R3*		Urotensin-2 Receptor-like	771154	*UTS2RL*	NA	NA
*UTS2R5*		Urotensin-2 Receptor-like	427799	*LOC42779*	100222682	*UTS2R5*

NA: not annotated.

What might be the potential significance of *UTS2B* expression in the songbird vocal control circuit? Is it expressed in a discrete cell population within HVC? Are urotensinergic receptors also present in HVC or its targets? Is *UTS2B* expression correlated with adult function of the circuit, or might it have some role in circuit development? To address these questions, we annotated the urotensinergic gene family in the zebra finch and used *in situ* hybridization (ISH) and immunohistochemistry to map *UTS2B* expression at the cellular level throughout the zebra finch brain, along with neuronal tract-tracing to determine the identity of *UTS2B*-expressing cells in HVC. We evaluated expression of the other main elements of the urotensinergic system (*UTS2* and the canonical urotensin receptors) and analyzed expression of *UTS2B* during the developmental period when HVC undergoes marked changes in connectivity and function in concert with changes in vocal/singing behavior. Finally, using data from the Allen Human Brain Atlas^32^, we assessed whether UTS2B expression might also occur in regions of the human brain involved in vocal control.

## Results

### Annotation of urotensinergic family genes in the zebra finch

As a first step in assessing the urotensinergic system in zebra finch, we reviewed the annotation of the peptides and their primary receptors in the zebra finch genome. Current public annotations of zebra finch genes (e.g., Ensembl, NCBI) reference a Sanger sequencing-based haploid genome assembly (Taeniopygia_guttata-3.2.4, GCA_000151805.2)^33^, but a preliminary phased diploid assembly using PacBio-based long-read sequencing is now available (Tgut_diploid_1.0, GCA_002008985.2). Though not yet annotated, the Tgut_diploid_1.0 assembly corrects significant gaps and misassemblies in the original reference^34^. However, we found that the two peptide precursor genes, *UTS2* (Entrez Gene 100222633) and *UTS2B* (Entrez Gene 100218300), were already correctly annotated in Taeniopygia_guttata-3.2.4; we confirmed their presence as single genes with two alleles each in Tgut_diploid_1.0 (Supplementary Annotation File).

**Figure 1:**
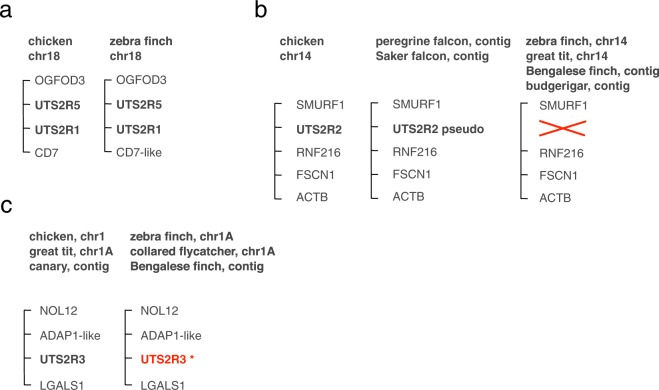
Syntenic relationships of urotensin receptor genes in birds, based on updated annotations from Table 1. (**a**) Chicken and zebra finch *UTS2R1* and *UTS2R5* are syntenic on chromosome 18. (**b**) *UTS2R2* is present on chromosome 14 in chicken. The syntenic locus is annotated as a pseudogene in falcons, and completely absent in zebra finch, great tit, Bengalese finch, and budgerigar. (**c**) *UTS2R3* is present and syntenic in chicken, great tit, and canary, but the syntenic loci in zebra finch, Bengalese finch, and collared flycatcher are disrupted by truncations or deletions and do not predict functional 7-transmembrane domain proteins (Fig. S1).

For the receptor genes, we found a more complex situation which was partially resolved by the new Tgut_diploid_1.0 assembly. The urotensin II receptor family has undergone a complex set of expansions and deletions in vertebrates (reviewed^35^) with some species (e.g., mammals) having only one *UTS2R* gene and others having as many as five. Four *UTS2R* genes have been identified in chicken: *UTS2R1*, *UTS2R2*, *UTS2R3*, and *UTS2R5*, according to the nomenclature of Tostivint and colleagues^35^ (Table 1), but only two were previously annotated in zebra finch. These genes are adjacent and syntenic on chromosome 18 in both chicken and zebra finch (Fig. 1). We reannotated both genes in the new phased diploid assembly using EST evidence from NCBI and published RNA-Seq data (Methods), obtaining additional 5′ coding sequence for *UTS2R5* and evidence for two alleles for each gene (Supplementary Annotation File). These genes encode proteins with 96% and 97% identity to chicken *UTS2R1* and *UTS2R5*, respectively. Based on this evidence we assigned the gene symbol for *UTS2R1* to Entrez Gene 100231339, and *UTS2R5* to Entrez Gene 100222682.

*UTS2R2*, missing from the taeGut-3.2.4 assembly, is also absent from the newer diploid assembly. Importantly, this is not due to missing sequence, as a gap in the predicted genomic locus (between SMURF1 and RNF216 on chromosome 14) is now completely filled in the newer assembly. Indeed, we could not find *UTS2R2* in existing annotations of any songbird or parrot, and the orthologous locus in the closely related Saker and peregrine falcons (Entrez Gene 106630916 and 106112120, respectively) is annotated as “gene type: pseudo” (Fig. 1). By BLAST analysis, the closest match to the falcon *UTS2R2* pseudogene sequence in the new diploid Tgut assembly is *UTS2R5*. Thus we conclude that *UTS2R2* was likely lost in this Australaves clade^36^.

The *UTS2R3* gene is annotated in great tit and canary as *UTS2*R-like, and syntenic with the chicken gene. However, the zebra finch locus, on chromosome 1A, encodes a truncated protein, lacking helices 4 to 7 of the 7 transmembrane helices typical of G-protein coupled receptors. Truncated proteins are also predicted by the genomic annotations for Bengalese finch (a near relative of zebra finch) and of collared flycatcher (Fig. 1). We carefully annotated two alleles of this gene in the Tgut_diploid_1.0 assembly, and both alleles encoded the same premature stop codon, while otherwise differing slightly; these genomic regions are sequenced at greater than 5X coverage with no gaps. We aligned whole genome sequencing reads from three wild and three domesticated zebra finches^37^ and all shared this premature stop codon. We also aligned RNA-Seq reads from five RA libraries^34^ and four whole brain libraries^38,39^ and observed in total only 7 mapped reads at this locus. Taken together, the extremely low expression and evidence for disruption of the coding sequence indicate that *UTS2R3* is not a viable candidate receptor for *UTS2B* in the brain. We thus focused on just *UTS2R1* and *UTS2R5* for further study of receptor RNA localization.

### Brain distribution of *UTS2B* in adult zebra finches

**Figure 2:**
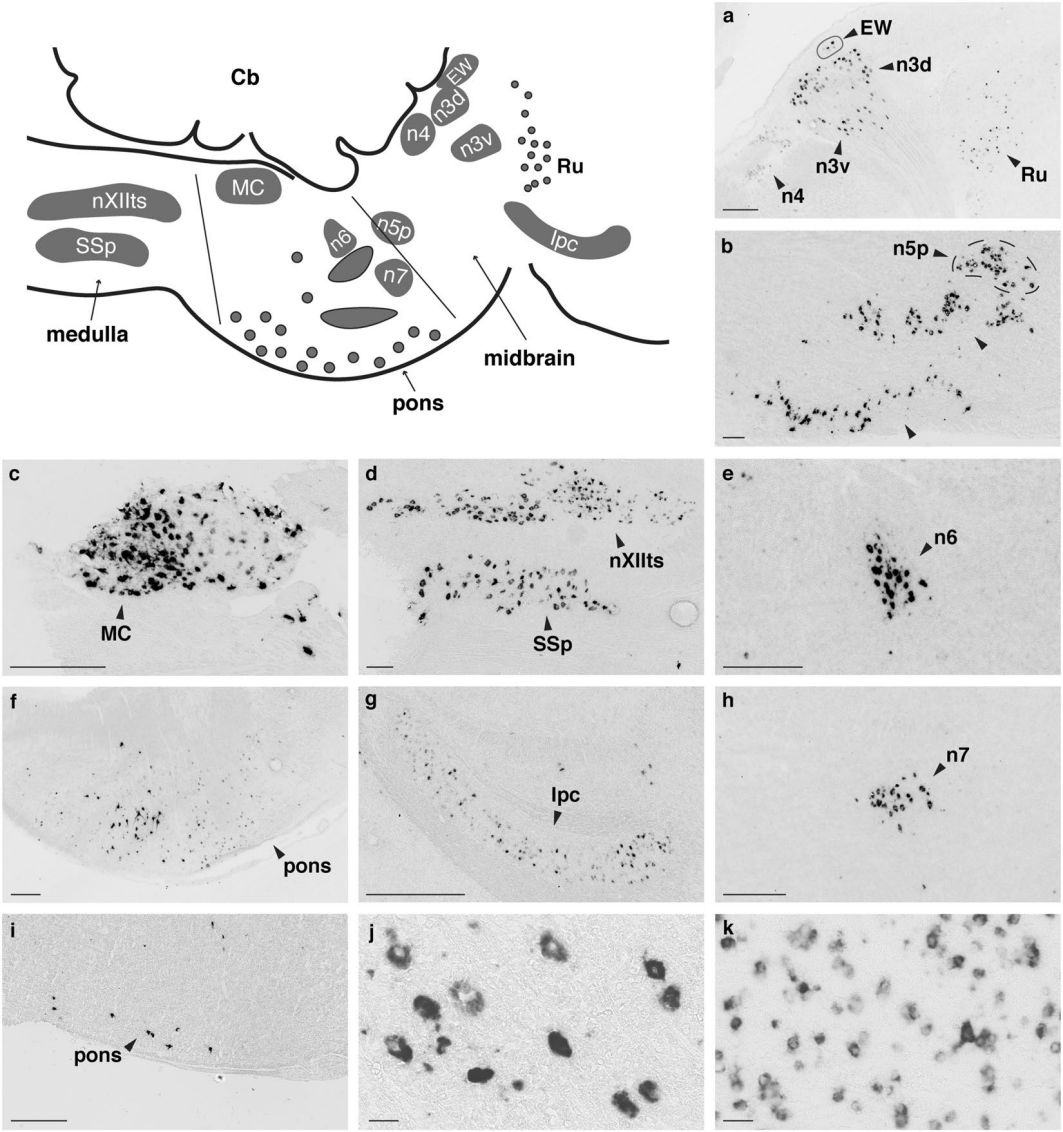
Brainstem *UTS2B* and *UTS2* gene expression. All photomicrographs are representative of male or female sagittal sections (no sex differences detected, number of observations is given in Table S1), showing *UTS2B* labelling (except *UTS2* in panel i). (**a**) The nucleus of Edinger-Westphal (EW), the dorsal part of the nucleus of the oculomotor nerve (n3d), the ventral part of the nucleus of the oculomotor nerve (n3v), the nucleus of the trochlear nerve (n4), the red nucleus (Ru); (**b**) The principal sensory nucleus of the trigeminal nerve (n5p), several unnamed pons nuclei; (**c**) the magnocellular nucleus, MC; (**d**) The tracheosyringeal motor nucleus (nXIIts), the supraspinal nucleus (SSp); (**e**) The nucleus of the abducens nerve, n6; (**f**) Scattered in the pons; (**g**) the parvocellular part of the isthmic nucleus, Ipc; (**h**) the nucleus of the facial nerve, n7; (**i**) *UTS2* expression in the pons; (**j,k**) Comparison of large *UTS2B* labelled cells in nXIIts [j] and smaller cells in HVC [k]. Scale bar = 250 μm (**a–i**) or 25 µm (**j,k**).

Using ISH with digoxigenin-labelled RNA probes, we mapped the expression of *UTS2B* RNA throughout the adult zebra finch brain, examining sections from 11 males and 16 females euthanized at midday. High densities of *UTS2B*-labelled cells were identified in several locations in the brainstem (Fig. 2), with no apparent sex differences. Labelled cells were especially abundant in motor nuclei of several cranial nerves, including the tracheosyringeal subdivision of the twelfth nerve nucleus (nXIIts, Fig. 2d), which innervates the syrinx and is thus directly involved in vocal control. We also detected brainstem cells expressing the *UTS2* paralog (Fig. 2i). Brainstem expression of both *UTS2* and *UTS2B* was detected early in juvenile development, within 12 h after hatching (Fig. S2).

**Figure 3:**
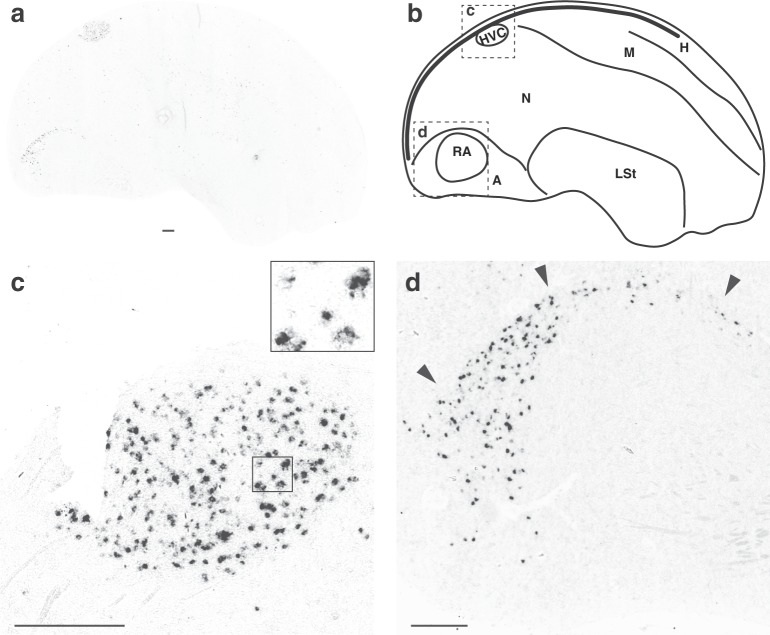
Male Pallium *UTS2B* gene expression. (**a**) *In situ* hybridization image of lateral sagittal section showing labelled cells scattered throughout the pallium, with concentrations of labelled cells dorsally in HVC and in a caudal portion of the arcopallium excluding RA (rostral to the right, dorsal up). (**b**) Line drawing of section in panel a labelling major landmarks (A – arcopallium; H – hyperpallium; LSt – lateral striatum; M –mesopallium; N – nidopallium; the lateral ventricle indicated by thick line running adjacent to caudal and dorsal borders); dashed lines indicate insets shown at higher magnification in panels c and d. (**c**) High *UTS2B* gene expression within cells localized in HVC. (**d**) high *UTS2B* gene expression within cells localized to a caudal portion of the dorsal arcopallium (A), adjacent to the robust nucleus of the arcopallium (RA). Arrowheads point to the pallial/sub-pallial lamina (LPS) running just dorsally to the area of cellular labelling. Number of observations is given in Table S1; scale bar = 500 μm.

In the pallium (i.e., cortical homolog), a high density of *UTS2B*-positive cells occurred in two regions in adults: nucleus HVC, but only in males (Fig. 3a,c); and an arc-like portion of the caudo-dorsal arcopallium outside RA, in both sexes (Fig. 3a,d). In male HVC, labelled cells of <25 µm diameter were observed throughout the extent of the nucleus (1.5–2.9 mm from the midline), varying in staining intensity (Figs 2k, 3c). Labelled cells were also detected scattered throughout the nidopallium and occasionally detected within the mesopallium, lateral striatum and subpallial regions (Fig. 3b). Labelled cells were absent in white matter, including laminae, commissures and major fibre tracts, as well as in blood vessels, choroid plexus, ventricular system and pia mater. In stark contrast to *UTS2B*, expression of *UTS2* was restricted to the brainstem (Fig. 2i).

**Figure 4:**
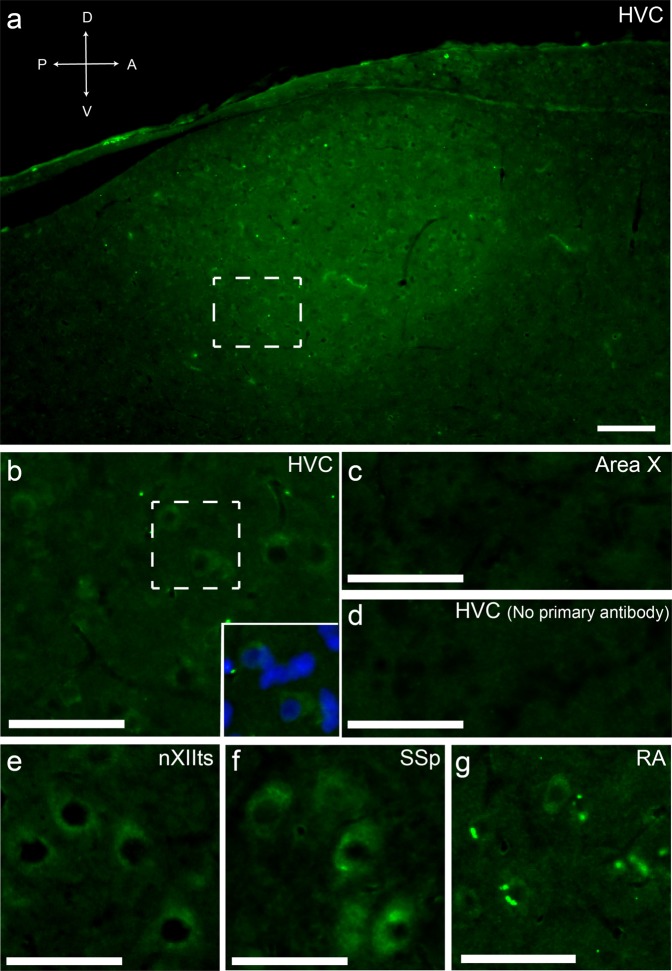
URP detection by immunohistochemistry. (**a**) Parasagittal section of caudal telencephalon showing increased immunostaining for URP in an oval area corresponding to nucleus HVC, immediately beneath the lateral ventricle. (**b**) Box inside HVC enlarged from panel a, showing cytoplasmic immunostaining pattern of a subset of cells (small inset, Hoechst counterstaining revealing all cell nuclei). (**c**) Field inside Area X, with negligible immunostaining. (**d**) Absence of primary antibody demonstrates negligible non-specific fluorescence (field inside HVC as in b). (**e**) Large labelled cells in brainstem nucleus nXIIts. (**f**) Large labelled cells in brainstem supraspinal nucleus (SSp). (**g**) Intense puncta of URP staining suggestive of synaptic terminals in RA. Bar = 100 µm (**a**) or 50 µm (**b–g**).

Using fluorescent immunohistochemistry with an antibody raised against the cyclic peptide CFWKYC (conserved in URP and UII of humans, rodents and birds) we detected clear evidence of a peptide within HVC (Fig. 4), which we interpret as URP given the robust expression of *UTS2B* RNA and the lack of *UTS2* RNA in HVC. As expected for a neuropeptide, signal was concentrated in cytoplasm of discrete labelled cells in HVC (Fig. 4b) and diffusely present in neuropil (Fig. 4a). With the exception of nucleus RA (Fig. 4g), we detected little or no labelling in areas lacking evidence of RNA expression, such as song nucleus Area X (Fig. 4c), and no non-specific signal (e.g., autofluorescence) when the primary antibody was omitted (Fig. 4d). Conversely, we detected intense immunostaining in cell bodies within brainstem nuclei where the RNA is abundant, such as nXIIts and SSP (Fig. 4e,f). In nucleus RA, despite the absence of mRNA expression, we observed small brightly labelled puncta suggestive of URP concentration in synaptic terminals (Fig. 4g).

### *UTS2B*-expressing cells in HVC project to song nucleus RA

**Figure 5:**
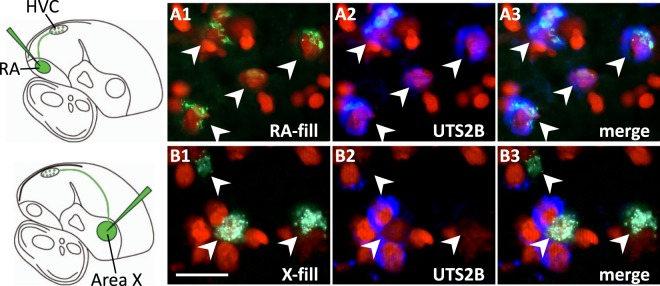
Selective expression of *UTS2B* in RA-, but not X-projecting HVC neurons. Schematics on the left depict injections of fluorescent tract-tracer (CTB-Alexa 488) into RA (top) or Area X (bottom), resulting in retrograde labelling of specific populations of projection neurons in HVC. (**a**) Retrogradely-labelled RA-projecting neurons (a_**1**_, green) co-localize with *UTS2B*-expressing cells (a_**2**_, blue), as confirmed by the merged image (a_3_). (**b**) Retrogradely-labelled X-projecting neurons (b_1_, green) do not co-localize with *UTS2B*-expressing cells (b_**2**_, blue), as confirmed by the merged image (b_**3**_). In all panels, arrowheads indicate location of individual backfilled neurons identified by characteristic donut-shaped cytoplasmic labelling that surrounds a nucleus (red, propidium iodide stain). *UTS2B* expression is revealed by fluorescent *in situ* hybridization with antisense riboprobes.

Functionally distinct cell classes in HVC can be defined by their projections. To determine which cell classes express *UTS2B*, we injected a fluorescent tract-tracer (CTB-Alexa 488) into the two major targets of HVC, RA and Area X, to retrogradely label RA- or X-projecting neurons in HVC (Fig. 5, schematics on left). We then examined co-localization with *UTS2B*-expressing cells using fluorescent *in situ* hybridization. The vast majority of the identified RA-projecting neurons (97%; n = 117 out of 121 retrogradely-labelled cells from 2 RA-injected birds) showed co-localization with *UTS2B* expression (Fig. 5a_1–3_), whereas only 4 cells showed tracer signal only. Furthermore, only 3 *UTS2B*-expressing cells in the fields examined showed no tracer deposit. Thus, essentially all *UTS2B*-expressing neurons in HVC correspond to RA-projecting neurons. In contrast, the vast majority of the identified X-projecting cells (97%; 64 out of 66 retrogradely-labelled cells from 2X-injected birds) showed no co-localization with *UTS2B*-expressing cells (Fig. 5b_1–3_), whereas in only two cells did the tracer appear to co-localize with *UTS2B* expression. Conversely, only 2 out of 178 *UTS2B*-expressing cells in the HVC sections examined appeared to show co-localization with the tracer signal from Area X. Thus, we conclude that *UTS2B* is selectively expressed in the subpopulation of HVC neurons that project to RA, and not in neurons that project to area X. Because this experiment seemed to account for all *UTS2B*-expressing cells in HVC, we deemed it unnecessary to perform double-labelling *in situs* with GAD2 to exclude expression in GABAergic cells.

### Brain distribution of Urotensin-2 Receptor transcripts

**Figure 6:**
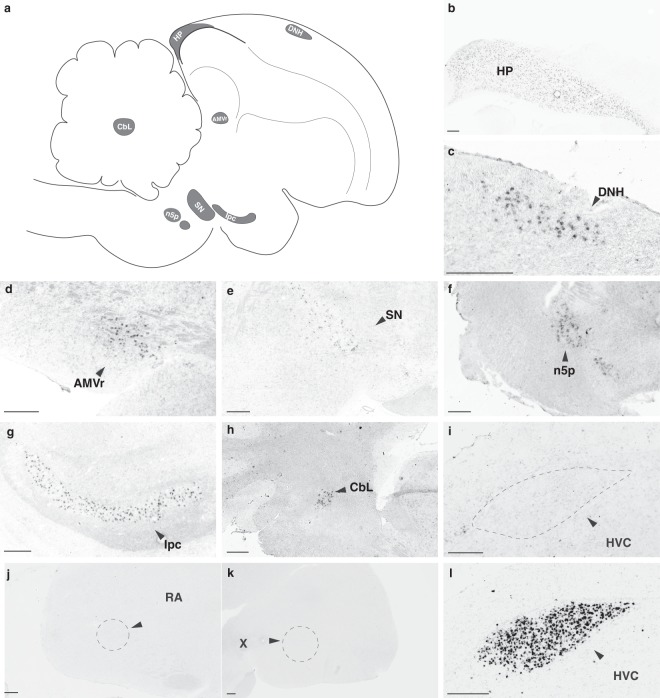
*UTS2R5* gene expression. *In situ* hybridization analysis. (**a**) Key for the figure, showing a schematic projection of labelled brain regions onto a 2-dimensional medial sagittal plane (note that these regions are distributed across multiple planes in the third medial-lateral dimension, not represented here); (**b**) labelling across the hippocampus (HP); (**c**) dorsal nucleus of the hyperpallium (DNH); (**d**) ventral medial arcopallium, rostral (AMVr); (**e**) substantia nigra (SN); (**f**) principal sensory nucleus of the trigeminal nerve (n5p); (**g**) the parvocellular part of the isthmic nucleus (Ipc); (**h**) the lateral nucleus of the cerebellum (CbL); (**i–l**) All four photomicrographs are from the same male that showed no visible *UTS2R5* gene expression in [**i**] HVC, [**j**] RA, [**k**] area X, while having [**l**] high *UTS2B* gene expression in HVC. Photomicrographs [**b–h**] are representative of males and females (no sex differences detected; number of observations is given in Table S1). Scale bar = 250 μm.

We mapped the brain expression of *UTS2R1* and 5 with ISH, using adjacent sections from the same series used to map *UTS2B* expression in Fig. 3. *UTS2R5* positive cells were labelled with less intensity in comparison to *UTS2B*, but high densities of *UTS2R5* cells were detected in various brain regions. These areas included the hippocampal formation (Fig. 6b) and the dorsal nucleus of the hyperpallium (DNH) (Fig. 6c); rostral part of the medio-ventral arcopallium (Fig. 6d); the substantia nigra (Fig. 6e); two brainstem nuclei (n5p, Ipc) in which *UTS2B* was also abundant (Fig. 6f,g); and the lateral cerebellar nucleus (Fig. 6h). The overall distribution was similar in males and females, with no apparent sex differences. Notably, we did not detect expression of *UTS2R5* within HVC itself (Fig. 6i), nor in the two song control nuclei that receive the projections from HVC (Fig. 6j,k). Lastly, we did not detect *UTS2R1* expression in brain tissue, although the same riboprobe detected *UTS2R1* expression in cardiac tissue (not shown). Scale bar = 20 µm.

### Early emergence of male-specific *UTS2B* expression in HVC

**Figure 7:**
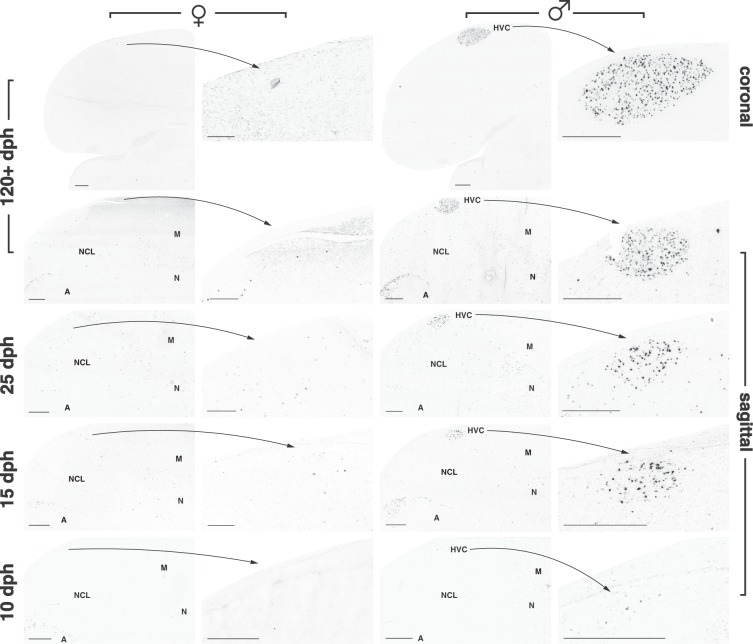
Developmental HVC *UTS2B* gene expression. Representative low power photomicrographs of the caudal forebrain in females (columns 1 and 2) and males (columns 3 and 4). Columns 2 and 4 show high power insets as indicated. Number of observations is given in Table S1; arcopallium – A; mesopallium - M; nidopallium - N; caudolateral nidopallium – NCL. Scale bars = 500 μm.

The evidence presented so far shows that *UTS2B* is highly expressed not only in brainstem nuclei (as in other vertebrates) but also in a key telencephalic nucleus of the oscine vocal control system, where it marks a specific subset of neurons – those that form the HVC-RA projection, which in the zebra finch is present only in adult males. This provided an opportunity to examine when this subset of neurons first emerges in development. We therefore used ISH to assess *UTS2B* in both sexes at four ages: post-hatch days 10 and 15, before formation of the projection to RA; day 25, when the projection from HVC to RA is beginning to develop and HVC is morphologically similar in males and females; and adults, after HVC has largely regressed in females^17^. At all juvenile ages and in both sexes, high densities of *UTS2B*-labelled cells were detected in the caudodorsal arcopallium, as well as low densities in the caudolateral nidopallium. However, only males showed high densities of labelled cells within HVC at any age (Fig. 7). Labelled cells were present in males in the vicinity of the presumptive HVC as early as 10 dph, and the cellular distribution indicates an oval shaped nucleus by 15 dph. Thus male-specific *UTS2B* expression emerges prior to morphological sexual differentiation, and prior to functional maturation of the nucleus.

### Evidence of UTS2B RNA expression in human inferior frontal cortex

On the basis of both functional criteria and connectivity, HVC has been considered analogous^5–8^ to the portions of human inferior frontal cortex implicated in human speech and singing^40^, comprising Brodmann areas 44 and 45, or respectively the opercular and triangular parts of the inferior frontal gyrus in the dominant (typically the left) hemisphere^41^. To assess whether UTS2B is expressed or enriched in these areas, we reviewed the microarray data for human brain gene expression made available through the Allen Brain Atlas^32^. Data are available for 55 sub regions of the frontal lobe, and UTS2B is annotated as enriched in three; one of the areas of enrichment is the opercular part of the right inferior frontal gyrus, although no enrichment is recorded for the left opercular or the triangular parts (Table S2).

## Discussion

Here, for the first time in an organism with vocal learning ability, we have assessed the brain expression and genome organisation of urotensin family peptide genes and their receptors. Surveying the distribution of the RNA, we found a conserved pattern of *UTS2B* expression in the brainstem as in other vertebrates, involving many of the same motor nuclei as in rodents^31^. In the forebrain, however, the expression pattern diverged sharply. In the zebra finch we saw scattered *UTS2B*-positive cells throughout the forebrain with high concentrations in two discrete areas, whereas in the mouse expression has been reported for only occasional cells in a few areas including hypothalamus^31^, with no areas of the mouse forebrain showing enriched local expression as we see in the zebra finch.

Of particular interest, we observed highly concentrated *UTS2B* expression in the vocal control nucleus HVC, where *UTS2B* specifically and comprehensively marked the male-specific neuronal subpopulation that projects to vocal control output nucleus RA. These neurons in HVC form the critical vocal pre-motor projection of the song system, arguably analogous to Broca’s area projection to laryngeal motor cortex^6^. They encode timing features that provide critical temporal cues for learned song^10,42^, undergo continuous replacement in adulthood^43–45^, and possess a characteristic electrophysiological signature that differentiates them from other specific populations in HVC^46^. Whether *UTS2B* contributes to any of these particular properties remains to determined.

A high density of *UTS2B*-expressing cells was also detected in the caudo-dorsal arcopallium in both sexes. A similar anatomical domain within arcopallium is defined by cholecystokinin (*CCK*) gene expression, although *CCK* and *UTS2B* expression patterns diverge outside the arcopallium (e.g., *CCK* RNA is absent from HVC)^47^. The avian arcopallium has complex functions and connections, and its internal organization is not yet well understood. In songbirds, portions of the arcopallium surrounding nucleus RA have been linked to the auditory pathways^48,49^ as well as to both courtship behaviour^50^ and song learning behaviour^51^, although such domains are respectively rostro-ventral and lateral rather than caudal or dorsal to RA. To our knowledge, this caudo-dorsal domain has not yet been investigated in any functional studies, thus a possible role for *UTS2B* expression in this region remains to be determined.

Taking advantage of recent improvements in the zebra finch genome assembly, we confirmed that there is a single *UTS2B* gene in zebra finch with a single paralog (*UTS2*) as in other vertebrates. Thus the novel expression of *UTS2B* in zebra finch telencephalon is not the result of avian lineage-specific gene duplication and divergence. Rather it must involve either an alternative pattern of *UTS2B* gene regulation, or alternative patterns of *UTS2B*-expressing cell survival and migration.

Using UTS2B expression as a cell type-specific developmental marker, we were able to obtain new insight into mechanisms that underlie the unusual development of the critical HVC-RA projection. This projection forms surprisingly late in brain development, and then only in males, with fibres entering RA from HVC ~25 days after hatching^17,52^. Yet by other measures (Nissl staining, gene expression) HVC is already present as a nucleus in both sexes two weeks before this^18–21^, and cells are already forming projections to Area X by 13 days post-hatch^18^. Both sexes initially appear to send fibres in the direction of RA, yet in females no fibres ever enter RA, and the Nissl-defined boundary of the female nucleus eventually recedes^17,52^. Our evidence now suggests a novel cellular explanation for both of these distinctive features, by revealing that the RA projection emerges from a discrete population of neurons that are found only in the male brain, even at early ages when the male and female HVC are superficially similar. It remains to be determined whether cells with equivalent potential are also present at this stage in the female brain (but have not differentiated to produce URP) or whether this represents a truly male-specific cell lineage. Exposure to estrogens in brain is necessary for formation of the projection in males^25^, and estrogen treatment in the first few weeks after hatching will masculinize the female HVC ultimately leading to a functional RA projection^24,53^. Thus we hypothesize that sex steroids in the brain must trigger the early production or differentiation of a discrete population of cells in HVC, which are marked by *UTS2B* expression and responsible for the eventual successful innervation of RA. It will be interesting to determine if early masculinizing estrogen treatment also induces *UTS2B* expression in the female HVC. The *UTS2B* gene itself may respond directly to sex steroids; in rodents, its expression is regulated in spinal motor neurons by androgen exposure^54^. It is also one of 17 genes that are commonly up-regulated in HVC in canaries, robins and white crowned sparrows after androgen treatment to promote singing and growth of HVC^29,30^.

What might be the function of *UTS2B* expression within the vocal control system? The *UTS2B* and *UTS2* genes encode cyclic peptides that share a disulphide-linked motif (Cys-Phe-Trp-Lys-Tyr-Cys) and are conserved across vertebrates^35^. The UII peptide has been studied the most with respect to behavioural actions, but URP likely has similar effects since they both act on the same receptor (reviewed in^55^ and^56^). Intracerebroventricular injections of UII in rats results in a gradual and long lasting increase in cerebral blood flow^57^, an increase in locomotor activity^58^ and anxiogenic behaviour^59^, and effects on blood pressure and stress responses^58,60,61^. We speculate that *UTS2B* expression in HVC, and release in RA, might conceivably link vocal activity to vascular regulation.

Consideration of *UTS2B* function and evolution also led us to assess the complement of urotensin receptor sequences expressed in zebra finch brain (Table 1, Fig. 1 and Supplementary Annotation file). Aided by an improved long-read assembly of the zebra finch genome as a reference^34^, we obtained missing 5′ sequence for *UTS2R5*, confirmed the orthology of *UTS2R1*, and annotated a previously unidentified locus for *UTS2R3* (which we classify as a pseudogene as the predicted peptide is truncated). The sole urotensin receptor in placental mammals is orthologous to *UTS2R1*, and is broadly expressed in the brain^56^. In zebra finch, we did not detect expression of *UTS2R1* in brain, whereas *UTS2R5* is expressed in brain with a similar broad pattern as the mammalian urotensin receptor. However, we also did not detect *UTS2R5* within HVC, RA or Area X. As we do not find evidence of expression of the canonical urotensinergic receptors within HVC or its targets, we suggest that urotensin expressed in HVC may exert its functions through somatostatin receptors which can interact with urotensin peptides e.g.^62^. The RNA for SSTR1, for example, is detectable (though not enriched) in HVC and its major targets RA and X^63^, and thus remains a possible mediator of UTS2B actions.

Intriguingly, reviewing the data for expression in the human brain available through the Allen Brain Atlas^32^, we found evidence for increased local expression of *UTS2B* RNA in Brodmann Area 44 (inferior frontal gyrus, opercular part, right side), which has been implicated in vocal production of melodic phrases and some aspects of prosody processing^40^. No enrichment is reported for the opercular or triangular parts of the left inferior frontal gyrus, which together are more typically considered as Broca’s area for speech and language generation. We speculate that this local enrichment in the right operculum could represent another example of convergent evolution of vocal control circuits in humans and songbirds^3^. The identification of UTS2B as a specific marker in songbird HVC may afford new experimental opportunities for monitoring and manipulating the development and function of a model vocal learning circuit.

## Materials and Methods

### Gene annotation

Manual annotation of urotensin receptor genes in the Tgut_diploid_1.0 long-read assembly^34^ was conducted using Apollo Genome Annotator v. 2.0.7^64^. Annotations were supported by mRNA sequences retrieved from NCBI EST database^65^ and aligned with exonerate version 2.3.0^66^ modified to support gff3 output (https://github.com/hotdogee/exonerate-gff3). Additional support was derived from RNASeq evidence: 5 libraries derived from zebra finch song nucleus RA^67^ (downloaded from http://gigadb.org/dataset/100311) and 4 brain libraries from NCBI Sequence Read Archive^68^ (SRA accession SRX738987, SRX1616454, SRX1616455, SRX1616463), aligned with HiSat2 version 2.1.0^69^. Genomic sequence for the premature stop codon in *UTS2R3* was confirmed by aligning genomic sequences from 3 wild and 3 domesticated individuals^37^ (ENA^70^ accession ERX1092185-ERX1092187 and ERX1092180-ERX1092182) with BWA-MEM version 0.7.17. BAM files were sorted and indexed with SAMtools version 1.6.

### Synteny analysis

Synteny comparisons are manually compiled from current annotations for chicken (Gallus_gallus-5.0, GCF_00002315.4, annotation release 104), zebra finch (Taeniopygia_guttata-3.2.4, GCF_000151805.1, annotation release 103, and manual annotation described as above), peregrine falcon (F_peregrinus_v1.0, GCF_000337955.1, annotation release 101), Saker falcon (F_cherrug_v1.0, GCF_000337975.1, annotation release 101), great tit (Parus_major1.1, GCF_001522545.2, annotation release 101), Bengalese finch (also known as white-rumped munia, LonStrDom1, GCF_002197715.1, annotation release 100), budgerigar (Melopsittacus_undulatus_6.3, GCF_000238935.1, annotation release 102), canary (SCA1, GCF_000534875.1, annotation release 101), and collared flycatcher (FicAlb1.5, GCF_000247815.1, annotation release 101), visualised with NCBI Genome Data Viewer v. 4.3^65^.

### Birds

In the UK, zebra finches (*Taeniopygia guttata*) were housed at Queen Mary University of London in large free flight social aviaries, kept on a 14 - hour light/10-hr dark cycle and were provided food and water *ad libitum*. Animal housing and welfare were in compliance with the European directives for the protection of animals used for scientific purposes (2010/63/EU). The Named Animal Care and Welfare Officer for Queen Mary University of London oversaw the housing and routine care. Experiments in UK were performed under Procedures Project License PPL70–8183. In USA, zebra finches were housed at OHSU following NIH guidelines for the use and care of animals in research and all procedures were approved by OHSU’s Institutional Animal Care Use Committee (IACUC).

### Surgery and tracer injection (USA)

Two adult male zebra finches were anesthetized by an intramuscular injection of 40 mg/kg Nembutal (Sodium pentobarbital), and then placed in a stereotaxic apparatus. In each bird, tracer injections were made into Area X in one hemisphere, and RA in the opposite hemisphere, with hemispheres alternated across birds, as follows. Feathers were removed from the head, and a midline incision to the skin was made to reveal the skull. Depending upon the injection target, a small craniotomy was made above the tracer injection site. Cholera Toxin Subunit B conjugated to Alexa Fluor 488 (CTB-Alexa; 1% in water), a retrograde tracer, was pressure injected (10–50 nL) through a glass pipette into either nucleus RA or Area X, depending upon the experiment. To maximize the number of backfilled cells in HVC, a series of large injections were made into each nucleus at different depths. Animals were allowed to survive for 4–5 days and then were euthanized by decapitation. Brains were quickly removed, blocked along the midline (parasagittal plane), and flash frozen in TissueTek O.C.T. Compound (Sakura Finetek) in a dry ice/isopropanol bath. Parasagittal (10 µm) brain sections were cut on a cryostat, thawed onto microscope slides (Superfrost plus; Fisher Scientific, Pittsburg, PA), and stored at −80 °C for *in situ* hybridization.

### Sex determination

The sex of juvenile animals taken prior to the age of external sexual differentiation was determined from blood taken post-mortem, by PCR targeting the sex-linked CHD (chromohelicase-DNA-binding) gene, which has different intronic lengths on the two sex chromosomes (Z and W)^71^.

### Tissue collection and processing

Bird were killed by decapitation, and whole brains were dissected, embedded in O.C.T compound, and rapidly frozen in a slurry of isopropanol and dry ice within 5 minutes. Tissues were stored at −80 °C prior to sectioning. Brains were sectioned in coronal or sagittal planes at 10 µm and collected onto Superfrost Plus slides and fixed with fresh 3% paraformaldehyde in PBS (pH 7.4) for 5 minutes. Slides were then rinsed in PBS (pH 7.4), dehydrated in an ascending ethanol series (70%, 95%, 100%; 2 min each), air dried, and stored at − 80 °C.

### Plasmid templates for riboprobe synthesis

A clone (Genbank: DV945629) corresponding to *UTS2B* was obtained from the ESTIMA zebra finch brain cDNA collection^72^. Templates to produce probes for other targets were cloned via reverse transcription-polymerase chain reaction (RT-PCR) using primers designed with Primer-BLAST^73^: *UTS2* (517 bps, Genbank: MH071741) forward primer GACACAACGGAACGTCCAATA and reverse primer CATAGAGTCTAAAGTTTGGCGCTG; *UTS2R5* (371 bps, Genbank: MH071743), forward primer GCAACTTCTACAGGTTCACGTC and reverse primer AGGATTTGTCCCGAACTGC; *UTS2R1* (517 bps, Genbank: MH071742), forward primer AGCAGTGGGTACTTCCAGAG and reverse primer ACAACTACAAATTTAGCACTGCTGC. Purified RT-PCR products were subcloned into TOPO-pCRII plasmids using the TOPO TA cloning kit (Life Technologies), using zebra finch whole brain RNA (Qiagen RNeasy Kit) as template. *UTS2R1* and *UTS2R5* probe sequences were aligned with Jalview 2.10.2b1^74^. All clones used for ISH were verified by BLAT or BLAST to align to a single locus in the zebra finch genome.

### *In Situ* Hybridisation (ISH)

Digoxygenin-(DIG)-riboprobes were synthesized and hybridized to brain sections using previously established protocols^75^. In brief, isolated plasmid DNA was restriction enzyme digested (BssHII, BamHI, or EcoRV; New England Biolabs; Ipswich, MA) to release the insert template, and twice purified with Qiagen’s PCR purification kit (Qiagen Inc., Valencia, CA) or GeneJet PCR purification kit (Thermo Fisher Scientific, Loughborough, UK). Antisense and sense probes were synthesized at 37 °C for 2 hours using the appropriate T3 or T7 RNA Polymerase (Promega, Madison, WI), and purified by Sephadex G-50 columns. Probes were applied to sections for overnight hybridization followed by washes, as described^75^. Hybridization specificity was confirmed by demonstration of contrasting patterns obtained with different probes, and absence of signal with omission of labelled probe. For further anatomical analysis, adjacent sections were counterstained using Hematoxylin and Eosin (H&E). All slides were coverslipped with Histomount (ThermoFisher Scientific).

### Imaging and Identification of DIG-labelled cells

ISH images were digitised with the NanoZoomer whole slide scanner (Hamamatsu, Japan). NDP.view2 software (Hamamatsu) was used to examine sections as well as to export TIFF images for figures. The ZEBrA finch brain atlas (Oregon Health & Science University, Portland, OR 97239; http://www.zebrafinchatlas.org) was used as a reference to identify annotated sub regions within the telencephalon (pallium, subpallium), diencephalon (thalamus, hypothalamus), mesencephalon (midbrain), metencephalon (pons, cerebellum), and myelencephalon (medulla) for all ISH sagittal sections, ranging 0–4.8 mm from the midline using the right or left hemispheres. Each section was compared to 1 of 18 reference sections in the ZEBrA Atlas in order to determine lateral distance from midline (0–4.8 mm) and the neuroanatomical name of the region. Cells were considered DIG-labelled if dark purple/brown circles (donut-shaped) were seen at high power indicating cytoplasmic labelling.

### Fluorescent *In situ* hybridization (FISH)

The method used for fluorescent ISH was essentially as described^75^ except the slides were developed for detection of fluorescent probes (Fig. 5). Briefly, slides were hybridized, washed and blocked^75^ for 30 min then incubated for 2 hours in TNB (100 mM Tris-HCl pH 7.4, 150 mM NaCl, 0.3% Triton X-100, 0.1% skim milk) with a horseradish peroxidase conjugated anti-DIG antibody (anti-DIG-HRP; 1:1500; Roche Applied Science). Slides were then washed 3 times for 5 min at RT in TNT (100 mM Tris-HCl pH9.5, 150 mM NaCl, 0.3% Triton X-100), and then incubated at room temperature for 1 hr with Alexa 350-conjugated-tyramide in amplification buffer (1:100 dil.; Invitrogen, Carlsbad, CA) according to the manufacturer’s recommended protocol. Slides were then washed 3 times for 5 min at RT in TNT, counterstained with propidium iodide (1 mg/ml in TNT), and coverslipped with Aquamount (Thermoscientific). Appropriate controls, including using sense strand probes or omitting riboprobes altogether were routinely incorporated to the hybridizations, and yielded no detectable signals.

### FISH and retrograde tracer co-localization

To assess the co-localization of *UTS2B* with HVC’s Area X- and RA-projecting cells we first Nissl stained brain sections obtained from the birds that received retrograde tracer injections into Area X or RA, and identified a range of brain sections that contained nucleus HVC (~1.7–2.7 mm from the midline). We next performed FISH using sections through HVC and antisense riboprobe for *UTS2B*. Co-localization of fluorescent retrograde tract tracer and *in situ* signal was assessed under high power (100X) using fluorescence optics on a Nikon E600 microscope and Neurolucida for cell mapping and counts. To assess co-localization, we identified individual retrogradely-labelled neurons based on the presence of characteristic “donut shaped” pattern of labelling, indicative of cytoplasmic distribution, that surrounded a nucleus (in red based on counterstaining with propidium iodide). Next, we assessed whether the *UTS2B*-expressing cells (blue *in situ* signal) co-localized with the retrogradely-labelled cells (green retrograde tracer signal). For each hybridization to a brain section obtained from an Area X or RA backfill we counted between 50–100 cells, and calculated the proportion of retrogradely-labelled cells that co-expressed *UTS2B*. High-power fluorescent photomicrographs (100X) were obtained using a Digital camera (DVC co., Austin, TX) coupled to a Nikon E600 microscope. Photoshop-CC 2018 (Adobe Systems Inc., San Jose, CA) was used to adjust the color, color balance, and contrast of photomicrographs. Figures were prepared in Illustrator CC 2018 (Adobe Systems Inc.) or Powerpoint 2017 (Microsoft).

### Immunohistochemistry for URP

The immunostaining was carried out similarly to previously described^76^. Briefly, naïve adult male zebra finches were deeply anaesthetised with sodium pentobarbital, then flushed with saline and perfused intracardially with 4% paraformaldehyde. After at least 2 days submerged in 20% sucrose, the left brain hemisphere was cut sagittally at 20 µm using a cryostat and thaw-mounted onto gelatinised coated slides. Antigen unmasking solution (H-3300, Vector lab) was used prior to overnight incubation with rabbit anti-urotensin II related peptide (1:200, H-071-17, Phoenix Pharmaceuticals, Inc) in a 10 mM phosphate-buffered saline solution containing 0.5% Triton X-100 and 0.05% sodium azide. The secondary antibody, donkey anti-rabbit Alexafluor 488 was incubated for 2 h before washed and coverslipped with Vectashield. Immunofluorescence was captured digitally on a Zeiss microscope using the HiPic 9 software.

### Assessment of expression data from Allen Brain Atlas

The human brain microarray dataset^32^ was downloaded using the Harmonizome resource^77^. The dataset provides the mean mRNA expression profiles for each of 17979 genes across 414 brain structures, derived from microarray analysis of 6 adult human brain sample sets. As described^32^, the structures are identified and classified using an anatomical ontology, and RNAs are identified as locally enriched in a particular area if the expression signal is at least 2-fold higher than measured in surrounding or complementary structures. We filtered the data to extract the 55 structures that comprise the frontal lobe, and present the results in Supplementary Table S2.

## Supplementary information

Supplemental Materials
