# Supplementary material for: Urotensin-related gene transcripts mark developmental emergence of the male forebrain vocal control system in songbirds
Source: Sci Rep. 2019 Jan 28;9:816. doi: 10.1038/s41598-018-37057-w (PMC6349858; doi:10.1038/s41598-018-37057-w)
Supplement: Supplementary file 1 — Supplemental Materials [file 41598_2018_37057_MOESM1_ESM.docx]

# SUPPLEMENTAL MATERIALS

# Urotensin-related gene transcripts mark developmental emergence of the male forebrain vocal control system in songbirds

Zachary W. Bell, Peter Lovell, Claudio Mello, Ping Yip, Julia M. George & David F. Clayton

- **Supplemental Figures**
  - **Figures S1-S2**
- **Supplemental Table S1 (number of observations)**
- **Supplemental Table S2 (*UTS2B* expression in frontal lobes, Allen Human Brain Atlas)**
- **Supplementary_annotation_file.gff3:**
  - Annotation of Urotensin Receptor genes, includes gff3 formatted annotation and predicted peptides for 2 alleles each of UTS2, UTS2B, UTS2R1, UTS2R5, and truncated UTS2R3 for the genome assembly Tgut_diploid_1.0 (GenBank accession GCA_002008985.2).


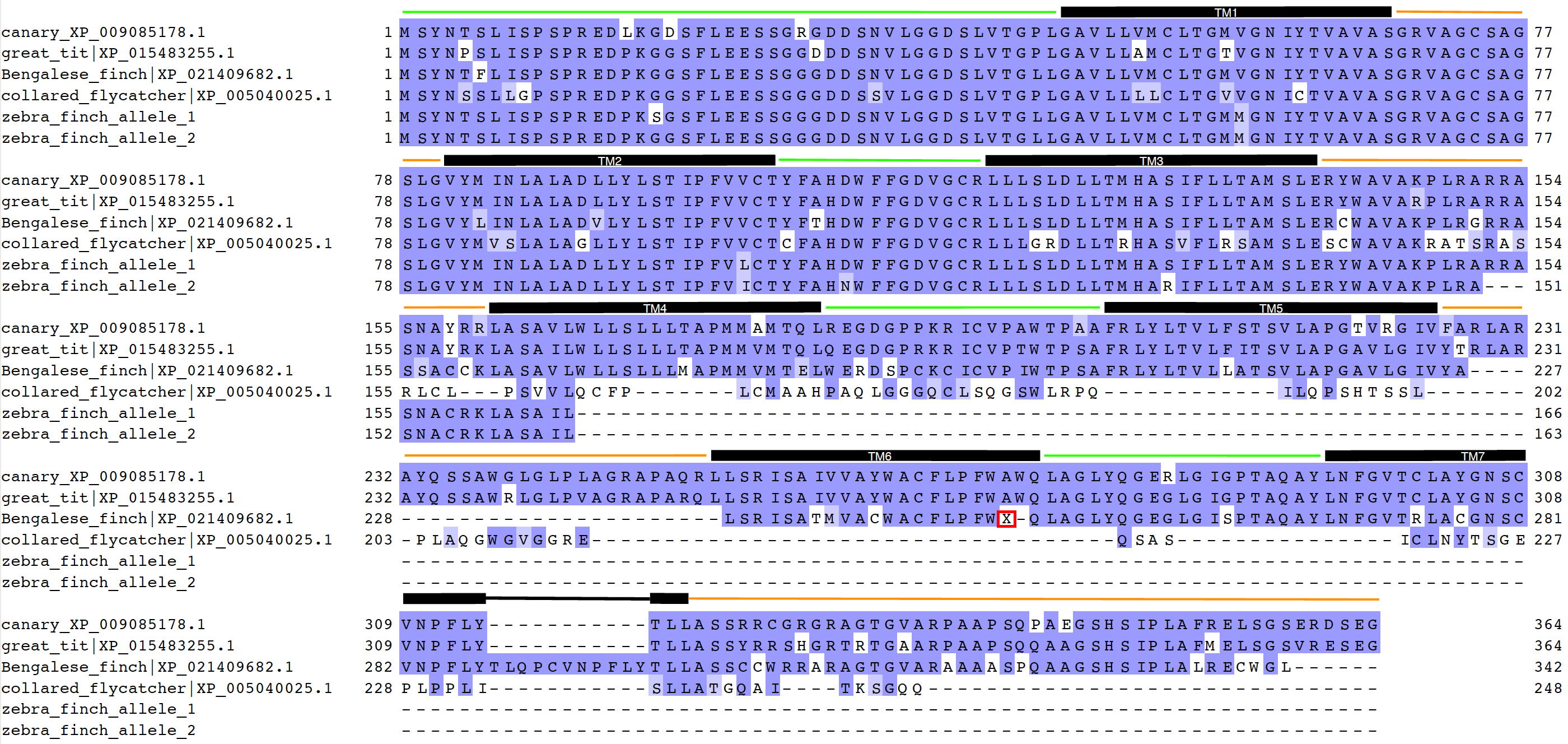


**Figure S1.**  **Alignment of predicted peptide sequences for songbird *UTS2R3*.** Canary and great tit sequences predict 7-transmembrane domain spanning proteins, as predicted for the G-protein coupled receptor family. The predicted peptide for Bengalese finch (XP_021409682.1) is missing the predicted internal loop between transmembrane helices 5 and 6, and contains a premature stop codon, while both collared flycatcher (XP_005040025.1) and zebra finch (our new annotation of Tgut_diploid_1.0) encode truncated proteins missing transmembrane helices 4-6. Black bars, transmembrane helices 1-7; green lines, predicted extracellular domains; orange lines, predicted intracellular domains; red box, in-frame stop codon.

**Figure S2. Developmental brainstem *UTS2* and *UTS2B* gene expression**. These photomicrographs are of midline sagittal brain sections of a male less than 12 hours after hatching. [A] H&E staining clearly shows the spinal cord (SC) and vertebrae (V), medulla-pons region, and the cerebellum (Cb). [B] Black arrows point to a small number of *UTS2* expressing cells (ISH) within the medulla-pons region (seen in 1 ♀, 3 ♂s). [C] Black arrows point to large pools of UTS2B expressing cells (ISH) throughout the SC and medulla-pons region (seen in 1♀ , 3 ♂s). Scale bar = 1 mm.

**Supplemental Table S1: Numbers of animals observed in Figs 2, 3, 6 and 7**

| Panel | Sex of image | #♀observed | # ♂ observed |
| --- | --- | --- | --- |
| 2a | ♂ | 12 | 4 |
| 2b | ♂ | 13 | 10 |
| 2c | ♀ | 10 | 5 |
| 2d | ♀ | 10 | 6 |
| 2e | ♀ | 14 | 7 |
| 2f | ♂ | 16 | 11 |
| 2g | ♂ | 6 | 7 s |
| 2h | ♂ | 15 | 11 |
| 2i | ♂ | 16 | 11 |
| 3 (overview) |  | 17 | 12 |
| 3a | ♂ | (absent in 17) | 12 |
| 3b | ♂ | 17 | 9 |
| 6a | ♀ | 14 | 6 |
| 6b | ♂ | 4 | 3 |
| 6c | ♂ | 8 | 5 |
| 6d | ♂ | 9 | 6 |
| 6e | ♀ | 2 | 4 |
| 6f | ♂ | 8 | 5 |
| 6g | ♂ | 3 | 2 |
| 7, 120+ dph |  | 11 | 9 |
| 7, 25 dph |  | 1 | 2 |
| 7, 15 dph |  | 4 | 2 |
| 7, 10 dph |  | 2 | 1 |

**Supplemental Table S2: *UTS2B* expression, Allen Human Brain Atlas data matrix** (Methods) filtered for *UTS2B* (gene ID 257313, probe set ID 1032754), and for all structures listed in the anatomy ontology under “Frontal Lobe”. Structures that comprise Broca’s area (inferior frontal gyrus opercular part, and inferior frontal gyrus triangular part) are highlighted. Expression values that are higher or lower by 2-fold or more are indicated by 1 or -1 respectively.

| **Structure** | **StructureID** | **Expression Value** |
| --- | --- | --- |
| middle frontal gyrus, left, inferior bank of gyrus | 4031 | 0 |
| middle frontal gyrus, left, superior bank of gyrus | 4030 | 0 |
| superior frontal gyrus, left, lateral bank of gyrus | 4024 | 0 |
| superior frontal gyrus, left, medial bank of gyrus | 4023 | 0 |
| frontal operculum, left | 4079 | 0 |
| paracentral lobule, anterior part, left, inferior bank of gyrus | 4074 | 0 |
| paracentral lobule, anterior part, left, superior bank of gyrus | 4073 | 0 |
| precentral gyrus, left, superior lateral aspect of gyrus | 4013 | 0 |
| precentral gyrus, left, inferior lateral aspect of gyrus | 4014 | 0 |
| precentral gyrus, left, bank of the central sulcus | 4015 | 0 |
| precentral gyrus, left, bank of the precentral sulcus | 4012 | 0 |
| inferior frontal gyrus, orbital part, left | 4045 | 0 |
| inferior frontal gyrus, opercular part, left | 4042 | 0 |
| inferior frontal gyrus, triangular part, left | 4039 | 0 |
| lateral orbital gyrus, left | 4060 | 0 |
| anterior orbital gyrus, left | 4054 | 0 |
| posterior orbital gyrus, left | 4057 | 0 |
| superior frontal gyrus, left | 4022 | 0 |
| paracentral lobule, anterior part, left | 4072 | 0 |
| superior rostral gyrus, left | 4898 | 0 |
| gyrus rectus, left | 4048 | 0 |
| medial orbital gyrus, left | 4051 | 0 |
| inferior rostral gyrus, left | 4901 | 0 |
| subcallosal cingulate gyrus, left | 4063 | 0 |
| parolfactory gyri, left | 4069 | 0 |
| posterior orbital gyrus, right | 4058 | 1 |
| frontal pole, right, inferior aspect | 4895 | -1 |
| frontal pole, right, superior aspect | 4894 | -1 |
| frontal pole, left, medial aspect | 4892 | -1 |
| inferior frontal gyrus, orbital part, right | 4046 | 0 |
| medial orbital gyrus, right | 4052 | 0 |
| inferior frontal gyrus, opercular part, right | 4043 | 1 |
| inferior rostral gyrus, right | 4902 | 0 |
| superior rostral gyrus, right | 4899 | 0 |
| frontal pole, right, medial aspect | 4896 | 0 |
| middle frontal gyrus, right, inferior bank of gyrus | 4034 | 0 |
| inferior frontal gyrus, triangular part, right | 4040 | 0 |
| precentral gyrus, right, bank of the precentral sulcus | 4017 | 0 |
| lateral orbital gyrus, right | 4061 | 0 |
| frontal operculum, right | 4080 | 0 |
| subcallosal cingulate gyrus, right | 4064 | 0 |
| paraterminal gyrus, right | 4067 | 0 |
| frontal pole, left, inferior aspect | 4891 | 0 |
| gyrus rectus, right | 4049 | 0 |
| superior frontal gyrus, right, lateral bank of gyrus | 4027 | 0 |
| anterior orbital gyrus, right | 4055 | 0 |
| middle frontal gyrus, right, superior bank of gyrus | 4033 | 0 |
| superior frontal gyrus, right, medial bank of gyrus | 4026 | 0 |
| frontal pole, left, superior aspect | 4890 | 0 |
| parolfactory gyri, right | 4070 | 1 |
| paracentral lobule, anterior part, right, inferior bank of gyrus | 4077 | 0 |
| paracentral lobule, anterior part, right, superior bank of gyrus | 4076 | 0 |
| precentral gyrus, right, superior lateral aspect of gyrus | 4018 | 0 |
| precentral gyrus, right, bank of the central sulcus | 4020 | 0 |
| precentral gyrus, right, inferior lateral aspect of gyrus | 4019 | 0 |

# SUPPLEMENTAL ANNOTATION FILE

Supplementary_annotation_file.gff3

##gff-version 3

##sequence-region MUGN01000909.1 1 4696417

MUGN01000909.1 . gene 4530263 4531975 . - . ID=gene_01;Name=UTS2R1_allele_01

MUGN01000909.1 . mRNA 4530263 4531975 . - . Parent=gene_01;ID=mRNA_01;Name=UTS2R1_allele_01

MUGN01000909.1 . exon 4530263 4531975 . - . Parent=mRNA_01

MUGN01000909.1 . CDS 4530688 4531836 . - 0 Parent=mRNA_01

###

##sequence-region MUGN01001167.1 1 643691

MUGN01001167.1 . gene 482470 484193 . - . ID=gene_02;Name=UTS2R1_allele_02

MUGN01001167.1 . mRNA 482470 484193 . - . Parent=gene_02;ID=mRNA_02;Name=UTS2R1_allele_02

MUGN01001167.1 . exon 482470 484193 . - . Parent=mRNA_02

MUGN01001167.1 . CDS 482906 484054 . - 0 Parent=mRNA_02

###

##sequence-region MUGN01000909.1 1 4696417

MUGN01000909.1 . gene 4448416 4450622 . - . ID=gene_03;Name=UTS2R5_allele_01

MUGN01000909.1 . mRNA 4448416 4450622 . - . Parent=gene_03;ID=mRNA_03;Name=UTS2R5_allele_01

MUGN01000909.1 . CDS 4449456 4450553 . - 0 Parent=mRNA_03

MUGN01000909.1 . exon 4448416 4450622 . - . Parent=mRNA_03

###

##sequence-region MUGN01001167.1 1 643691

MUGN01001167.1 . gene 402386 404582 . - . ID=gene_04;Name=UTS2R5_allele_02

MUGN01001167.1 . mRNA 402386 404582 . - . Parent=gene_04;ID=mRNA_04;Name=UTS2R5_allele_02

MUGN01001167.1 . exon 402386 404582 . - . Parent=mRNA_04

MUGN01001167.1 . CDS 403425 404522 . - 0 Parent=mRNA_04

###

##sequence-region MUGN01000779.1 1 12254580

MUGN01000779.1 . gene 2667416 2667919 . + . ID=gene_05;Name=UTS2R3_fragment_allele_01

MUGN01000779.1 . mRNA 2667416 2667919 . + . Parent=gene_05;ID=mRNA_05;Name=UTS2R5_partial

MUGN01000779.1 . exon 2667416 2667919 . + . Parent=mRNA_05

MUGN01000779.1 . CDS 2667416 2667916 . + 0 Parent=mRNA_05

###

##sequence-region MUGN01002868.1 1 7822023

MUGN01002868.1 . gene 1690873 1691408 . - . ID=gene_06;Name=UTS2R3_fragment_allele_02

MUGN01002868.1 . mRNA 1690873 1691408 . + . Parent=gene_06;ID=mRNA_06;Name=UTS2R3 partial

MUGN01002868.1 . CDS 1690917 1691408 . + 0 Parent=mRNA_06

MUGN01002868.1 . exon 1690873 1691408 . + . Parent=mRNA_06

###

##sequence-region MUGN01000053.1 1 4882880

MUGN01000053.1 . gene 2495370 2499190 . + . ID=gene_07;Name=UTS2_allele_01

MUGN01000053.1 . mRNA 2495370 2499190 . + . Parent=gene_07;ID=mRNA_07;Name=UTS2_allele_01

MUGN01000053.1 . exon 2495370 2495504 . + . Parent=mRNA_07

MUGN01000053.1 . exon 2498916 2499190 . + . Parent=mRNA_07

MUGN01000053.1 . exon 2498284 2498327 . + . Parent=mRNA_07

MUGN01000053.1 . exon 2497703 2497804 . + . Parent=mRNA_07

MUGN01000053.1 . CDS 2495402 2495504 . + 0 Parent=mRNA_07

MUGN01000053.1 . CDS 2497703 2497804 . + 2 Parent=mRNA_07

MUGN01000053.1 . CDS 2498284 2498327 . + 2 Parent=mRNA_07

MUGN01000053.1 . CDS 2498916 2499032 . + 0 Parent=mRNA_07

###

##sequence-region MUGN01001396.1 1 2758046

MUGN01001396.1 . gene 1206523 1210341 . + . ID=gene_08;Name=UTS2_allele_02

MUGN01001396.1 . mRNA 1206523 1210341 . + . Parent=gene_08;ID=mRNA_08;Name=UTS2_allele_02

MUGN01001396.1 . exon 1206523 1206657 . + . Parent=mRNA_08

MUGN01001396.1 . exon 1210067 1210341 . + . Parent=mRNA_08

MUGN01001396.1 . CDS 1206555 1206657 . + 0 Parent=mRNA_08

MUGN01001396.1 . CDS 1208854 1208955 . + 2 Parent=mRNA_08

MUGN01001396.1 . CDS 1209434 1209477 . + 2 Parent=mRNA_08

MUGN01001396.1 . CDS 1210067 1210183 . + 0 Parent=mRNA_08

MUGN01001396.1 . exon 1209434 1209477 . + . Parent=mRNA_08

MUGN01001396.1 . exon 1208854 1208955 . + . Parent=mRNA_08

###

##sequence-region MUGN01000039.1 1 1440326

MUGN01000039.1 . gene 90212 99396 . - . ID=gene_09;Name=UTS2B_allele_01

MUGN01000039.1 . mRNA 90212 99396 . + . Parent=gene_09;ID=mRNA_09;Name=UTS2B_allele_01

MUGN01000039.1 . exon 95579 95616 . + . Parent=mRNA_09

MUGN01000039.1 . exon 96933 97026 . + . Parent=mRNA_09

MUGN01000039.1 . exon 92613 92714 . + . Parent=mRNA_09

MUGN01000039.1 . exon 90212 90436 . + . Parent=mRNA_09

MUGN01000039.1 . exon 98759 99396 . + . Parent=mRNA_09

MUGN01000039.1 . CDS 90313 90436 . + 0 Parent=mRNA_09

MUGN01000039.1 . CDS 92613 92714 . + 2 Parent=mRNA_09

MUGN01000039.1 . CDS 95579 95616 . + 2 Parent=mRNA_09

MUGN01000039.1 . CDS 96933 97026 . + 0 Parent=mRNA_09

MUGN01000039.1 . CDS 98759 98784 . + 2 Parent=mRNA_09

###

##sequence-region MUGN01002153.1 1 405336

MUGN01002153.1 . gene 36059 45409 . + . ID=gene_10;Name=UTS2B_allele_01

MUGN01002153.1 . mRNA 36059 45409 . + . Parent=gene_10;ID=mRNA_10;Name=UTS2B_allele_02

MUGN01002153.1 . exon 42857 42950 . + . Parent=mRNA_10

MUGN01002153.1 . exon 38535 38636 . + . Parent=mRNA_10

MUGN01002153.1 . CDS 36241 36364 . + 0 Parent=mRNA_10

MUGN01002153.1 . CDS 38535 38636 . + 2 Parent=mRNA_10

MUGN01002153.1 . CDS 41498 41535 . + 2 Parent=mRNA_10

MUGN01002153.1 . CDS 42857 42950 . + 0 Parent=mRNA_10

MUGN01002153.1 . CDS 44684 44709 . + 2 Parent=mRNA_10

MUGN01002153.1 . exon 36059 36364 . + . Parent=mRNA_10

MUGN01002153.1 . exon 41498 41535 . + . Parent=mRNA_10

MUGN01002153.1 . exon 44684 45409 . + . Parent=mRNA_10

###

##FASTA

>UTS2R1_allele_01_predicted

MSLSDELESHFSATPYMVTDTSEDSVFRIRPNASANATGDGVWAAGSTEDMIAICTIGAILSLMCVVGVT

GNVYTLLVMCHYLRSSASMYIYIINLALADLLYLLTIPFIVGTYFIQKWYFGDVGCRILFSLDFLTMHAS

IFTLTVMSTERYFAVLKPLDTVKRSKSYRKAIAVLIWLVSLLLTLPMLIMIQLVQRDNKSICLPTWSKLS

YKVYLTILFGTSIVGPGVVIGYLYIRLAKIYWVSQTASFKQTKRLPNQKVLYLIFTIVLVFWACFLPFWI

WQLLFQYYESFPLSPKVMKNINYLTTCLTYSNSCINPFLYTLLTKNYREYLKNRQRSLSSSSGYFQRRNR

FQRISGRSLSTSSQHCTETYVLAHAPLGNSSA

>UTS2R1_allele_02_predicted

MSLSDELESHFSATPYMVTDTSEDSMFRIRPNASANATGDGAWAAGSTEDMIAICTIGAILSLMCVVGVT

GNVYTLLVMCHYLRSSASMYIYIINLALADLLYLLTIPFIVGTYFIQKWYFGDVGCRILFSLDFLTMHAS

IFTLTVMSTERYFAVLKPLDTVKRSKSYRKAIAVLIWLVSLLLTLPMLIMIQLVQRDNKSICLPTWSKLS

YKVYLTILFGTSIVGPGVVIGYLYIRLAKIYWVSQTASFKQTKRLPNQKVLYLIFTIVLVFWACFLPFWI

WQLLFQYYESFPLSPKVMKNINYLTTCLTYSNSCINPFLYTLLTKNYREYLKNRQRSLSSSSGYFQRRNR

FQRISGRSLSTSSQHCTETYVLAHAPLGNSSA

>UTS2R5_allele_01_predicted

MEPNGTAAAGDNGTAAAAAAAAPGGGPLLIPSAFGTVLSVMYVAGVAGNVYTLVVMCHSARCAAPMYSSI

VSLALADLLYLSTIPFIVCTYLAQDWYFGDLGCRILLSLDLLTMHASIFTLTLMCTERYLAVTRPLDTLK

RSRGYRKVTAGAVWSVSLLLTLPMMLMVTLTEGGKAEGKVKRMCAPTWSVDAYRTYLTVLFSTSIMAPGI

IIGFLYTRLARTYLESQRNPPHKEKSKRSPRQKVLIMIFSIVLVFWACFLPFWIWQLVRLYSSSLQLTTQ

TQKCINYLVTCLTYSNSCINPFLYTLLTKNYREYLRNRHRNFYRFTSSFRKRGSNLQCSWGRSMSSSNQY

DYSSEALGMATLKDK

>UTS2R5_allele_02_predicted

MEPNGTAAAGDNGTAAAAAAAAPGGGPLLIPSAFGTVLSVMYVAGVAGNVYTLVVMCHSARCAAPMYSSI

VSLALADLLYLSTIPFIVCTYLAQDWYFGDLGCRILLSLDLLTMHASIFTLTLMCTERYLAVTRPLDTLK

RSRGYRKVTAGAVWSVSLLLTLPMMLMVTLTEGGKAEGKVKRMCAPTWSVDAYRTYLTVLFSTSIMAPGI

IIGFLYTRLARTYLESQRNPPHKEKSKRSPRQKVLIMIFSIVLVFWACFLPFWIWQLVRLYSSSLQLTTQ

TQKCINYLVTCLTYSNSCINPFLYTLLTKNYREYLRNRHRNFYRFTSSFRKRGSNLQCSWGRSMSSSNQY

DYSSEALGMATLKDK

>UTS2R3_fragment_allele_01_predicted

MSYNTSLISPSPREDPKSGSFLEESSGGGDDSNVLGGDSLVTGLLGAVLLVMCLTGMMGNIYTVAVASGR

VAGCSAGSLGVYMINLALADLLYLSTIPFVLCTYFAHDWFFGDVGCRLLLSLDLLTMHASIFLLTAMSLE

RYWAVAKPLRARRASNACRKLASAIL

>UTS2R3_fragment_allele_02_predicted

MSYNTSLISPSPREDPKGGSFLEESSGGGDDSNVLGGDSLVTGLLGAVLLVMCLTGMMGNIYTVAVASGR

VAGCSAGSLGVYMINLALADLLYLSTIPFVICTYFAHNWFFGDVGCRLLLSLDLLTMHARIFLLTAMSLE

RYWAVAKPLRASNACRKLASAIL

>UTS2_allele_01_predicted

MHKLILCCLIIVSFSCPLLSLPIINASEMSYQHPADEDSRLNLERLGSTSLLQLLPELLGTLTEDSRTGL

TPSNYNPGENIKETFHGNHPRNAFLGRFLIKDRKQYKKRGNLSECFWKYCV

>UTS2_allele_02_predicted

MNKLILCCLIIVSFSCPLLSLPIINASEMSYQHSADEDSRLNLERLGSTSLLQLLPELLGTLTEDSRAGL

TPSNYNPGENIKETFHGNHPRNAFLGRFLIKDRKQYKKRGNLSECFWKYCV

>UTS2B_allele_01_predicted

MLLGSGNVEKMWSAQLCLGVLTILTMALCVPSTHGDPFLLQENRVLPEREDTNHENTLLTLLLNKKFAWR

RPESIDWELAKKFEELEELEKLKDQLSAEDGSEVAYALESLSASQPKKRACFWKYCI

>UTS2B_allele_02_predicted

MLLGSGNVEKMWSAQLCLGVLTILTMALCVPSTHGDPFLLQENRVLPEREDTNHENTLLTLLLNKKFAWR

RPESIDWELAKKFEELEELEKLKDQLSAEDGSEVAYALESLSASQPKKRACFWKYCI
